# Supplementary material for: Summer Is Coming! Tackling Ocean Warming in Atlantic Salmon Cage Farming
Source: Animals (Basel). 2021 Jun 16;11(6):1800. doi: 10.3390/ani11061800 (PMC8234874; doi:10.3390/ani11061800)
Supplement: Supplementary file 1 [file animals-11-01800-s001.zip › animals-1160351-supplementary.pdf]

Table S1 Thermal windows for life-cycle stages of Atlantic salmon (*Salmo salar*) reported in the literature.

Life stage	Thermal window (°C)			Literature
	Optimum range	Minimum	Maximum	
**Egg**		0	16	[1]
**Egg/Alevin**	4 to 7.2	0.5	12 to 23	[1-4]
**Fry**	8 to 19	0.5	23.5 to 28	[5, 6]
**Parr**	8 to 19	0	27 to 33	[1, 7-9]
**Smolt**	7 to 14	5	28	[10-12]
**Adult**	2-8 to 13-19	0 to 8	23 to 25	[7, 13, 14]

Table S2 Thermal windows for life-history events reported in the literature**.**

Life-history event	Thermal window (°C)			Literature
	Optimum range	Minimum	Maximum	
**Spawning**	5 to 8	1.5	10 to 12	[15]
**Smoltification**	Around 10	3	15	[10, 16-18]
**Migration to sea**	Around 10 to 11	5.8 to 11	20	[10, 19, 20]
**Migration to stream**		5 to 8	16 to 23	[13, 21]

References

1. Elliott, J.; Elliott, J. Temperature requirements of Atlantic salmon *Salmo salar*, brown trout *Salmo trutta* and Arctic charr *Salvelinus alpinus*: predicting the effects of climate change. *Journal of Fish Biology* **2010,** 77, (8), 1793-1817.

2. DeCola, J.N. *Water quality requirements for Atlantic salmon, USDI.* Boston, USA, 1970.
3. Peterson, R.H.; Spinney, H.C.E.; Sreedharan, A. Development of Atlantic salmon (*Salmo salar*) eggs and alevins under varied temperature regimes. *Journal of the Fisheries Research Board of Canada* **1977,** 34, (1), 31-43.
4. Ojanguren, A.F.; Reyes-Gavilan, F.G.; Munoz, R.R. Effects of temperature on growth and efficiency of yolk utilisation in eggs and pre-feeding larval stages of Atlantic salmon. *Aquaculture International* **1999,** 7, (2), 81-87.
5. Peterson, R.H.; Sutterlin, A.M.; Metcalfe, J.L. Temperature preference of several species of *Salmo* and *Salvelinus* and some of their hybrids. *Journal of the Fisheries Research Board of Canada* **1979,** 36, (9), 1137-1140.
6. Grande, M.; Andersen, S. Critical thermal maxima for young salmonids. *Journal of Freshwater Ecology* **1991,** 6, (3), 275-279.
7. DFO Temperature threshold to define management strategies for Atlantic salmon (Salmo salar) fisheries under environmentally stressful conditions. *Canadian Science Advisory Secretaria* **2012**.
8. Elliott, J. Tolerance and resistance to thermal stress in juvenile Atlantic salmon, Salmo salar. *Freshwater Biology* **1991,** 25, (1), 61-70.
9. Elliott, J.; Hurley, M. A functional model for maximum growth of Atlantic salmon parr, Salmo salar, from two populations in northwest England. *Functional Ecology* **1997,** 11, (5), 592-603.
10. McCormick, S.D.; Hansen, L.P.; Quinn, T.P.; Saunders, R.L. Movement, migration, and smolting of Atlantic salmon (Salmo salar). *Canadian Journal of Fisheries and Aquatic Sciences* **1998,** 55, (S1), 77-92.
11. Nilsson, J.; Moltumyr, L.; Madaro, A.; Kristiansen, T.S.; Gåsnes, S.K.; Mejdell, C.M.; Gismervik, K.; Stien, L.H. Sudden exposure to warm water causes instant behavioural responses indicative of nociception or pain in Atlantic salmon. *Veterinary and Animal Science* **2019,** 8, 100076.
12. Johnston, C.E.; Saunders, R.L. Parr–smolt transformation of yearling Atlantic salmon (*Salmo salar*) at several rearing temperatures. *Canadian Journal of Fisheries and Aquatic Sciences* **1981,** 38, (10), 1189-1198.
13. Shepard, S.L. *Atlantic salmon spawning migrations in the Penobscot River, Maine: fishways, flows and high temperatures*. University of Maine, USA, 1995.

14. Strom, J.F.; Thorstad, E.B.; Rikardsen, A.H. Thermal habitat of adult Atlantic salmon Salmo salar in a warming ocean. *Journal of Fish Biology* **2020,** 96, (2).
15. Heggberget, T.G. Timing of spawning in Norwegian Atlantic salmon. *Canadian Journal of Fisheries and Aquatic Sciences* **1988,** 45, (5), 845-849.
16. Wedemeyer, G.A.; Saunders, R.L.; Clarke, W.C. Environmental factors affecting smoltification and early marine survival of anadromous salmonids. **1980**.
17. McCormick, S.D.; Moriyama, S.; Bjornsson, B.T. Low temperature limits photoperiod control of smolting in Atlantic salmon through endocrine mechanisms. *American Journal of Physiology-Regulatory Integrative and Comparative Physiology* **2000,** 278, (5), R1352-R1361.
18. Handeland, S.O.; Wilkinson, E.; Sveinsbo, B.; McCormick, S.D.; Stefansson, S.O. Temperature influence on the development and loss of seawater tolerance in two fast-growing strains of Atlantic salmon. *Aquaculture* **2004,** 233, (1-4), 513-529.
19. Whalen, K.G.; Parrish, D.L.; McCormick, S.D. Migration timing of Atlantic salmon smolts relative to environmental and physiological factors. *Transactions of the American Fisheries Society* **1999,** 128, (2), 289-301.
20. Thorstad, E.B.; Whoriskey, F.; Uglem, I.; Moore, A.; Rikardsen, A.H.; Finstad, B. A critical life stage of the Atlantic salmon *Salmo salar*: behaviour and survival during the smolt and initial post-smolt migration. *Journal of Fish Biology* **2012,** 81, (2), 500-542.
21. Jonsson, B.; Jonsson, N.; Hansen, L.P. Factors affecting river entry of adult Atlantic salmon in a small river. *Journal of Fish Biology* **2007,** 71 (4), 943-956.
